# Wheat cleistogamy is expressed by the dosage effect of homoeologous *AP2* mutant alleles carrying a point mutation in the miR172 target site

**DOI:** 10.1270/jsbbs.25078

**Published:** 2026-06-23

**Authors:** Katsuyuki Kakeda, Agetha Bigie Nanape, Hinako Ogawa, Miyoko Nitta

**Affiliations:** 1 Graduate School of Bioresources, Mie University, 1577 Kurimamachiya-cho, Tsu, Mie 514-8507, Japan; 2 Faculty of Bioresources, Mie University, 1577 Kurimamachiya-cho, Tsu, Mie 514-8507, Japan

**Keywords:** *Triticum aestivum*, floret closing, lodicule, *Apetala2*, *cly1*, microRNA172, additive effect

## Abstract

Cleistogamy, or closed flowering, has received significant attention in wheat (*Triticum aestivum*) due to its wide range of applications. A stable cleistogamy system in barley (*Hordeum vulgare*), regulated by a single gene (*cly1*), has been successfully utilized in practical breeding. The aim of this study was to establish stable cleistogamy in hexaploid wheat by combining point mutations in the microRNA172 target site of three *AP2* homoeologs, which are the wheat orthologs of barley *Cly1*. Triple mutants carrying the three relevant mutant *AP2* alleles successfully exhibited cleistogamy resulting from suppression of lodicule swelling. Further investigation revealed that strong mutant alleles from two homoeologous loci (*AP2-B* and *AP2-D*) were necessary and sufficient to induce cleistogamy equivalent to that in barley, which stably suppressed lodicule development. The analysis also demonstrated that the total dosage of mutant *AP2* alleles contributed to lodicule suppression and cleistogamy, likely correlating with the gene expression levels. Approaches for resolving the compact spike trait, which is expressed simultaneously with cleistogamy and is disadvantageous in practice, are also discussed.

## Introduction

The primary type of flowering in grasses is open flowering, also known as chasmogamy. Swelling of the lodicules is the main force that opens the florets. This pushes the palea and lemma apart during the anthesis. The suppression of lodicule swelling results in closed flowering, which is also known as cleistogamy. The genetic basis of cleistogamy caused by lodicule developmental deficiency has been well-studied in rice (*Oryza sativa*) (Lombardo *et al.* 2017, Ohmori *et al.* 2012, Yoshida *et al.* 2007a) and barley (*Hordeum vulgare*) (Nair *et al.* 2010, Turuspekov *et al.* 2004, Wang *et al.* 2015, 2021). Barley cleistogamy is determined by a single gene located at the *Cleistogamy 1* (*cly1*) locus on chromosome 2H. This gene encodes a barley ortholog of the *Arabidopsis* Apetala 2 (AP2) transcription factor HvAP2. A point mutation in the microRNA172 (miR172) target site of the recessive natural variant alleles (*cly1.b* and *cly1.c*) causes cleistogamy. This mutation inhibits miR172 binding to its mRNA, thereby allowing expression of the HvAP2 protein. HvAP2 negatively regulates lodicule development. In practice, cleistogamous barley cultivars carrying the *cly1.b* allele reduce the risk of Fusarium head blight (FHB), possibly by preventing the fungus from entering and infecting the florets (Yoshida *et al.* 2005, 2007b). Consequently, there has been increased focus on applying the barley cleistogamy system to wheat breeding.

In this context, we attempted to induce cleistogamy in hexaploid wheat (*Triticum aestivum*). While screening for induced point mutations in three *AP2* homoeologs, which are the orthologs of the barley *Cly1* gene (Ning *et al.* 2013a, 2013b), we identified point mutations in two of the three homoeologs, *AP2-A* and *AP2-D*. However, single mutants did not exhibit cleistogamy as anticipated for hexaploids (Nanape *et al.* 2023). Subsequently, we found that accumulating these mutations significantly reduced anther extrusion, but did not result in cleistogamy (Nanape *et al.* 2024). We initially hypothesized that cleistogamous wheat could be engineered by combining the relevant *AP2* mutant alleles from all three homoeologous loci into a single plant. However, it remains unclear whether the point mutation in the *AP2-B* homoeolog is necessary to achieve cleistogamy. A recent study of wheat spikelet development identified a relevant point mutation in the *AP2-B* homoeolog (Debernardi *et al.* 2020), which can be introduced. Therefore, the aim of this study was to test our initial hypothesis by generating triple mutants homozygous for point mutations in the three *AP2* homoeologs. We then investigated the flowering phenotype and lodicule development. We also analyzed the dosage effect of individual mutant alleles on lodicule suppression to achieve cleistogamy.

## Materials and Methods

### Plant materials

Table 1 lists the parental genotypes and abbreviations used to produce the two triple mutants. The Japanese winter wheat cultivar ‘Kitahonami’ is the donor of single *AP2* mutants carrying the induced mutant alleles *AP2-A1*, *AP2-D1* and *AP2-D2* (see Supplemental Fig. 1; Nanape *et al.* 2023). Near-isogenic lines (NILs) of these BC_3_ progeny, obtained by backcrossing with Kitahonami, were intercrossed to produce two double mutants: *AP2-A1/AP2-D1* and *AP2-A1/AP2-D2* (Nanape *et al.* 2024). The Australian winter wheat cultivar ‘Wedgetail’ is the donor of the mutant *AP2-B* allele, which carries a point mutation in the miR172 target site (Supplemental Fig. 1). This allele was originally identified in a dwarf compact spike mutant and was named *rAP2l-B2* by Debernardi *et al.* (2020). Original mutant seeds were obtained from the Commonwealth Scientific and Industrial Research Organization (CSIRO). For simplicity, we refer to the *rAP2l-B2* allele as *AP2-B2*. We determined the entire genomic DNA sequence of the *AP2-B2* allele (DDBJ, accession no. LC897307) using the sequencing protocol described by Nanape *et al.* (2023) to confirm that there were no mutations other than that at miR172.

In the present study, we crossed two double mutants, A1D1 and A1D2, with the single *AP2-B* mutant, B2, to screen for two triple mutants homozygous for the three mutant alleles. The F_1_ plants resulting from reciprocal crosses (A1D1 × B2 and A1D2 × B2) were then self-pollinated to produce the F_2_ progeny. After genotyping approximately 300 plants from each F_2_ population, we screened 7 and 5 plants for the A1B2D1 and A1B2D2 triple-mutant genotypes, respectively (Table 1). We also obtained the F_3_ progeny from self-pollinating the two-triple mutant F_2_ plants.

Two F_2_ populations were generated to study the dosage effects of the mutant alleles. The first population was obtained through a reciprocal cross between the single mutants *AP2-B2* and *AP2-D2*. The latter used the BC_3_ progeny. This population, F_2_ (B2 × D2), was segregated into nine genotypes (Table 2). The second population, F_2_ (A1D1 × B2), was obtained from the same cross used to screen for the triple mutant A1B2D1 as described above. All segregants were completely genotyped for the three homoeologs, resulting in 24 of 27 possible genotypes (Table 2). In these two F_2_ populations, each genotype was abbreviated to include only the mutant alleles. This made it easier to determine the total number of mutant alleles required for the dosage comparison.

Plants were grown in a field at Mie University as described by Nanape *et al.* (2024). The survey was conducted primarily in the spring of 2022 (two triple mutants of the F_2_ generation) and spring of 2023 (two triple mutants of the F_3_ generation and two F_2_ populations). Triple mutants in later generations (F_4_ and F_5_) were observed for further confirmation in 2024 and 2025.

### Genotyping and quantitative PCR (qPCR) assays

Genotyping of the three *AP2* homoeologs was performed using allele-specific PCR to amplify and distinguish wild-type and mutant alleles in genomic DNA. The protocols for *AP2-A* and *AP2-D* homoeologs have been previously described by Nanape *et al.* (2024). Allele-specific amplification of the *AP2-B* homoeolog was performed by using a modified protocol described by Debernardi *et al.* (2020). Primers and PCR cycling parameters are listed in Supplemental Table 1.

Total RNA was extracted from immature spikes at the terminal spikelet or white anther stage (Kirby and Appleyard 1981) and subjected to qPCR to examine the transcript abundance of the three *AP2* homoeologs. All procedures and analytical protocols were performed as described by Nanape *et al.* (2023).

### Phenotypic and histological analysis of lodicules, as well as other phenotypic assessments

Lodicule size was measured using the first and second florets at anthesis as described by Nanape *et al.* (2023). This study focused on the lodicule width (LW) and depth (LD) as key indicators of floret opening and closing. Cross sections of the lodicules were prepared using florets during anthesis. After removing the lemma, the floret was fixed in a sodium phosphate buffer solution containing 10% formalin (pH 7.2) and stored at 4°C. Standard paraffin-embedding and microtome-sectioning procedures were used to produce 10 μm-thick serial sections on glass slides. Sections were deparaffinized and stained with toluidine blue.

Chasmogamous (CH) and cleistogamous (CL) flowering was primarily determined by visual inspection in the field based on the presence or absence of extruded anthers on the spikes at anthesis. The complete suppression of anther extrusion (AE) was defined as CL flowering. In addition to normal CH flowering, we occasionally encountered CH that exhibited highly suppressed AE and were classified as (CH). For a quantitative assessment, we calculated the AE rate per spike as described by Nanape *et al.* (2024). We also calculated the rachis internode length for spike density, as described by Nanape *et al.* (2023). The data were analyzed using the Tukey–Kramer HSD test to detect significant differences between multiple comparisons (*P* < 0.05).

## Results

### The two triple mutants exhibit cleistogamy

The triple mutants A1B2D1 and A1B2D2 exhibited cleistogamy in the F_2_ generation (Table 1, Fig. 1A). Anther extrusion (AE) was completely suppressed (Fig. 2A). Morphologically, they were notable for their extremely dense (compact) spikes due to significant reductions in rachis internode length (Supplemental Fig. 2A). The single mutant B2, the common parent of both triple mutants, exhibited CH flowering (Fig. 1A) with a reduced AE rate of 10.5% (Fig. 2A). This reduction was more pronounced than that observed in the two parental double mutants, A1D1 (68.4%) and A1D2 (33.5%), although the latter genotype was previously reported to exhibit the lowest AE rate among the mutants (Nanape *et al.* 2024). The F_3_ progeny of both triple mutants inherited CL flowering. However, F_3_ plants with the A1B2D1 genotype exhibited a slightly higher, albeit very low, AE rate of 0.83% (Fig. 2B), compared to the AE rate of 0.14% in F_2_ plants (Fig. 2A). Nevertheless, this value fell within the range of AE rates obtained from the examination of various CL barley cultivars (data not shown). The A1B2D2 mutant showed virtually no AE in the F_3_ progeny (Fig. 2B). Both triple mutants maintained a compact spike morphology in the F_3_ progeny; however, this was more variable than that in the F_2_ plants (Supplemental Fig. 2B).

### Both triple mutants undergo lodicule swelling suppression necessary for cleistogamy

The floral organs of both the triple mutants appeared normal, except the lodicules (Fig. 1B). At anthesis, the lodicules of the two triple mutants differed from those of the other genotypes in terms of swelling (Figs. 1C, 3A). A comparison of lodicule sizes revealed significant differences in lodicule width (LW) and depth (LD) between the two triple mutants (F_2_ plants) and the five other parental genotypes (Fig. 2A). Furthermore, the A1B2D2 lodicules were significantly smaller than the A1B2D1 lodicules in terms of both LW and LD. These trends in lodicule size were confirmed in the F_3_ progeny of both triple mutants (Fig. 2B). A scattergram of LD and AE clearly showed a correlation between them (Fig. 2C). However, the AE rates were not completely consistent with the lodicule size, which was particularly evident for B2. Additionally, scattergrams of LW and LD for individual F_2_ and F_3_ plants illustrate the size differences between the two triple mutants (Supplemental Fig. 3A, 3B). The F_3_ plants exhibited greater variation in lodicule size than the F_2_ plants, possibly because of their broader genetic backgrounds, resulting from the two distant parent cultivars.

Histological analysis of the lodicule sections revealed clear differences in lodicule development between the two triple mutants (Fig. 3B). Serial-section lodicule images of the two triple mutants are shown in Supplemental Fig. 4. Notably, the exceptionally small lodicules observed in the A1B2D2 mutant were primarily due to the absence of cellular regions where swelling normally occurs, as observed in other genotypes. The remaining portions of the A1B2D2 lodicules consisted of fewer sparse cells with no vascularization. These findings are consistent with those of typical CL barley lodicules (Nair *et al.* 2010, Wang *et al.* 2015, 2021). In contrast, the A1B2D1 lodicules had vascularized cellular regions that corresponded to the swollen area, although these regions were smaller than those observed in the other five CH genotypes. However, cells within the A1B2D1 lodicules were less organized than those within the CH genotypes. Vascularization was unevenly distributed, as seen more clearly between two lodicules within the same floret (see Supplemental Fig. 4 for details). These findings suggest that, despite their decent size, the A1B2D1 lodicules are partially abnormal or dysfunctional in terms of swelling.

### Mutant alleles required for cleistogamy

Two F_2_ populations with segregated mutant alleles were analyzed to determine which alleles were necessary and sufficient for cleistogamy. The first F_2_ population (B2 × D2) was examined to determine the dosage effect of the *AP2-B2* (*B^2^*) and *AP2-D2* (*D^2^*) alleles and to assess the impact of the absence of the *AP2-A1* (*A^1^*) allele. A field survey revealed that the nine genotypes segregated into six CH and three CL phenotypes (Table 2). The CL genotypes contained either three (*B^2^D^2^D^2^* and *B^2^B^2^D^2^*) or four (*B^2^B^2^D^2^D^2^*) mutant alleles. All these genotypes were confirmed to have an AE rate of virtually zero (Supplemental Fig. 5A). A comparison of lodicule size revealed a significant reduction in both LW and LD between the three CL genotypes and the other genotypes (Fig. 4A). A scattergram of LW and LD also shows a gradual decrease in the size of the lodicules from *B^2^D^2^D^2^* to *B^2^B^2^D^2^* and *B^2^B^2^D^2^D^2^* (Fig. 4B). Although the differences in lodicule size among the three CL genotypes were not entirely significant, the *B^2^B^2^D^2^D^2^* genotype had the lowest mean values for both LW and LD. These values were equivalent to those of the triple mutant A1B2D2 (Fig. 2A, F_2_ generation). These findings suggest that three *B^2^* and *D^2^* alleles are sufficient for cleistogamy expression, and that four alleles could suppress lodicules as effectively as six, with the addition of two *A^1^* alleles.

The second F_2_ population (A1D1 × B2) was used to determine the contribution of the three mutant alleles to the production of the triple mutant, A1B2D1. Due to the limited number of F_2_ plants, the *A^1^A^1^B^2^B^2^D^1^D^1^* genotype could not be obtained this time. Of the 24 genotypes obtained and examined, only two (*A^1^A^1^B^2^B^2^D^1^* and *A^1^B^2^B^2^D^1^D^1^*) exhibited the CL phenotype (Table 2). Another genotype carrying five mutant alleles (*A^1^A^1^B^2^D^1^D^1^*) was classified as CH. Three genotypes (*A^1^B^2^B^2^*, *A^1^B^2^B^2^D^1^* and *B^2^B^2^D^1^D^1^*) were classified as (CH) because they exhibited more suppressed AE than other CH genotypes. Examination of the AE rate revealed that five genotypes carrying two *B^2^* alleles and one or more additional *A^1^*or *D^1^* alleles (*A^1^*, *D^1^*, *A^1^D^1^*, *A^1^A^1^D^1^* or *A^1^D^1^D^1^*) exhibited significantly greater AE suppression than other genotypes (shown in red in Supplemental Fig. 5B). These genotypes tended to have smaller lodicules (red in Supplemental Fig. 6). Consequently, six genotypes carrying two *B^2^* alleles were selected for testing, with *B^2^B^2^* as the control, to determine the significant differences in lodicule size (Fig. 5A). Although not entirely significant, the lowest mean LW and LD values were found in *A^1^B^2^B^2^D^1^D^1^*, which was consistent with the lowest AE rate. However, the LW and LD values were higher than those obtained for the triple mutant A1B2D1 (Fig. 2A). Additionally, comparison of lodicule sizes among the nine genotypes carrying one *B^2^* allele may provide insights into the additive effects of the *A^1^* and *D^1^* alleles (Fig. 5B). Similar trends were observed for LW and LD. Having additional *A^1^* and/or *D^1^* alleles along with a single *B^2^* allele contributes to varying degrees of reduction in lodicule size. Significant differences were especially evident for LD compared to the *B^2^* control genotype. In summary, these results suggest that the *A^1^* and *D^1^* alleles act additively with the two essential *B^2^* alleles to promote cleistogamy in the triple mutant A1B2D1.

### Transcriptional profiling of mutant alleles

qPCR analysis was used to compare the transcript abundances of the three *AP2* homoeologs among the six genotypes, including the two CL triple mutants (A1B2D1 and A1B2D2), using RNA from immature spikes (Fig. 6). The transcription levels of the three wild-type alleles in the WT(WDG) differed from those in the WT(KH). In particular, the *AP2-A* transcript abundance was exceptionally low in the WT(WDG), presumably because of minimal transcription. Similar levels were confirmed in the B2 mutant derived from the WT(WDG), although the reason for this is unclear. Compared to WT(KH), WT(WDG) exhibited more abundant *AP2-B* transcripts, particularly at the white anther stage, and fewer *AP2-D* transcripts at both stages. The B2 mutant exhibited remarkably high transcript levels of the *AP2-B2* allele at both stages. This is in contrast to the modest increase in *AP2-D2* transcript levels observed in the D2 mutant. The two CL triple mutants also exhibited higher levels of *AP2-B2* transcripts, particularly at the white anther stage. These levels were comparable to those of the B2 mutant at the white anther stage but much lower at the terminal spikelet stage. The transcript levels of the other two mutant alleles (*AP2-A1* and *AP2-D1* or *D2*) in the two triple mutants were similar to or lower than those of the wild-type alleles in WT(KH). These results suggest that the expression levels of mutant alleles may be influenced by their interactions with coexisting mutant alleles.

## Discussion

### The two cleistogamous triple mutants have distinctive, suppressed lodicules

Nanape *et al.* (2023, 2024) reported that the three mutant *AP2* alleles obtained from the *AP2-A* and *AP2-D* homoeologs affect lodicule swelling and anther extrusion differently, depending on the type of point mutation in the miR172 target site (Supplemental Fig. 1). The *AP2-A1* and *AP2-D1* alleles, which carry the same point mutation, produce similar outcomes. In contrast, the *AP2-D2* allele has a significantly greater effect. These differences could be explained by variations in the predicted interaction strength between miR172 and the target sites of the mutant alleles. Based on its predicted interaction with miR172, the newly introduced *AP2-B2* allele is presumed to excert a strong effect (Nanape *et al.* 2024). Indeed, the *AP2-B2* mutant has been reported to have smaller lodicules and a 30% reduction in swollen areas (Debernardi *et al.* 2020). However, none of the previously reported *AP2* mutants in wheat exhibit cleistogamy. A primary challenge in achieving wheat cleistogamy is the requirement for three sets of homozygous *AP2* mutant alleles, as initially hypothesized. The present study demonstrated that the two triple mutants, A1B2D1 and A1B2D2, became CL due to the suppression of lodicule swelling (Fig. 1). However, the degree of lodicule suppression differed between the two mutants.

The A1B2D2 mutant had significantly smaller lodicules than the A1B2D1 mutant (Fig. 2), primarily due to the stronger effect of the *AP2-D2* allele. The differences were in size and development. Histological analysis of the cross sections revealed that the A1B2D2 lodicules lacked cellular regions that typically become vascularized and swell with hydration toward anthesis (Fig. 3). Additionally, the A1B2D2 lodicules appeared lamina-like when removed from the ovary. Preliminary observations also showed that the A1B2D2 lodicules did not swell upon exposure to 2,4-D, similar to those in CL barley (Wang *et al.* 2015, 2021). These significant developmental defects in the lodicules are consistent with those of CL barley lodicules carrying *cly1.b* or *cly1.c* (Nair *et al.* 2010, Wang *et al.* 2015). These findings strongly suggested that the A1B2D2 mutant exhibited complete cleistogamy, comparable to that of barley. In contrast, the A1B2D1 mutant exhibited partially swollen lodicules with varying degrees of swelling among the plants and/or florets. Cross sections of the swollen areas were less vascularized with fewer cells than those of the other CH genotypes (Fig. 3). A similar phenomenon occurs in *cly1.b2* of barley. Although it is CL in the field, it can undergo open flowering when treated with 2,4-D (Wang *et al.* 2015). This was due to the partial swelling of the lodicule in response to 2,4-D. Cross sections showed that the *cly1.b2* lodicules have more organized and vascularized swollen areas, even at the green anther stage preceding anthesis. These areas differ from those in the A1B2D1 lodicules. We also observed that the A1B2D1 mutant maintained floret closure when treated with 2,4-D. However, unlike the A1B2D2 mutant, the A1B2D1 mutant exhibited increased, yet unstable, lodicule swelling in response to 2,4-D teatment (data not shown). Therefore, it is likely that the A1B2D1 lodicules have developmental defects that permit partial swelling, but are insufficient to open the floret.

### The total dosage of mutant *AP2* alleles contributes to cleistogamy in hexaploid wheat

Further investigation revealed that the homozygote for the two strong mutant alleles, *AP2-B2* and *AP2-D2* (*B^2^B^2^D^2^D^2^*), exhibited cleistogamy equivalent to that of the A1B2D2 triple mutant (Fig. 4). This finding indicates that none of the three homoeologous *AP2* mutant alleles are essential for cleistogamy in wheat. In addition, cleistogamy was enabled by three mutant alleles (*B^2^B^2^D^2^* and *B^2^D^2^D^2^*), whereas two alleles (*B^2^B^2^*, *B^2^D^2^* and *D^2^D^2^*) were insufficient. These results demonstrate that cleistogamy occurs through the additive effects of the mutant *AP2* alleles, which quantitatively suppress lodicule swelling. Analyses of segregating genotypes for *AP2-A1*, *AP2-B2* and *AP2-D1* also supported the dosage effects of the mutant alleles. Cleistogamy was observed in the five-allele mutant genotypes (*A^1^A^1^B^2^B^2^D^1^* and *A^1^B^2^B^2^D^1^D^1^*), which require two *AP2-B2* alleles (Table 2). However, these two mutant genotypes exhibited weaker lodicule suppression than the triple mutant A1B2D1, which carried all six mutant alleles (Figs. 2A, 5A). Additional effects on reducing lodicule depth were observed for the *AP2-A1* and *AP2-D1* alleles in genotypes carrying a single *AP2-B2* allele (Fig. 5B). These findings suggest that the two weak mutant alleles contribute to cleistogamy by additively affecting lodicule suppression. Based on these results, we conclude that the total dosage of mutant *AP2* alleles contributes to cleistogamy in hexaploid wheat.

A dosage effect has also been reported for another wheat *AP2-like* gene, *Q* (*AP2L-A5Q*). The *Q* gene is a well-known wheat domestication gene that carries a point mutation in the miR172 target site compared with the wild-type *q* allele (*AP2L5-Aq*) (Simons *et al.* 2006). Muramatsu (1963) proposed the concept of the quantitative effects of *Q* (*q*) genes based on the analysis of chromosome 5A addition lines in ‘Chinese Spring’ aneuploids. For example, the spike phenotype gradually changes with the dosage of *Q* (*q*) genes, which corresponds to the number of introduced 5A chromosomes. This results in spike types ranging from long speltoid to normal squarehead, short subcompact and extreme compact. A similar dosage effect was also observed for the *AP2-A1* and *AP2-D1/D2* alleles, which reduced rachis internode length, resulting in denser, more compact spikes (Nanape *et al.* 2024). A similar dosage mechanism can be applied to spike type (rachis internode development) and lodicule size (lodicule development), as demonstrated in the present study. The effect of dosage on the individual mutant *AP2* allele may be attributed to its expression level. Given the remarkably high transcript abundance of the *AP2-B2* allele, it is reasonable to conclude that this mutant allele plays a critical role in inducing cleistogamy. However, lodicule suppression in barley cleistogamy is thought to depend more on the translational level of the *cly1* gene than on its transcriptional level (Anwar *et al.* 2018). Furthermore, the regulation of homoeologous gene expression in hexaploid wheat might be more complicated, particularly when interactions between miR172 and its duplicated target genes are involved. Currently, it is difficult to quantify the effect of individual *AP2* mutant allele dosages on lodicule development based solely on transcript abundance.

### Comparative views on wheat cleistogamy and future perspectives in breeding

Previous studies have reported several other CL wheats, including the Chinese accession U24 (Kubo *et al.* 2010, 2013) and the induced mutant ZK001 (Tang *et al.* 2019, 2020). The genes responsible for these CL phenotypes remain unknown. However, as Nanape *et al.* (2024) have suggested, the genetic mechanisms underlying cleistogamy likely differ from those demonstrated in this study. U24 exhibits fully swollen lodicules with significantly suppressed filament elongation. ZK001, which was obtained from a single mutagenesis event, has abnormal lodicules that do not fully swell. Nevertheless, the LW reduction rate at anthesis was only 24% in ZK001 relative to the parental cultivar YM18, as calculated based on measurements by Tang *et al.* (2020). This contrasts with the approximately 60% reduction rate observed in A1B2D2 in the present analysis. Despite these differences, cleistogamy in both U24 and ZK001 has been reported to increase resistance against FHB (Kubo *et al.* 2010, Tang *et al.* 2019, 2020). A similar advantage has been observed in CL barley cultivars (Yoshida *et al.* 2005, 2007b). However, because this trait does not confer true resistance to the pathogen, it is still necessary to spray pesticides to prevent disease when immature kernels are ripening and the remaining anthers emerge from the florets (Yoshida *et al.* 2008). In the present study, it was difficult to precisely assess the effects of CL traits on FHB resistance in the triple mutants because the Australian parental cultivar ‘Wedgetail’ is highly susceptible to FHB under current growing conditions, whereas the other parent, ‘Kitahonami’, is a resistant cultivar from Japan. Consequently, many genetic factors affecting resistance, besides floret opening, were likely segregated to varying degrees among the progeny obtained from the two distant cultivar-derived mutants. Despite visual inspection under pesticide-free conditions, the triple mutants appeared to exhibit an intermediate level of susceptibility between the two cultivars. Further precise evaluation of FHB resistance with near-isogenic genetic backgrounds is necessary.

Mutant *AP2* genotypes that exhibit complete cleistogamy in hexaploid wheat always have compact spikes due to pleiotropic effects that reduce the rachis internode length (Supplemental Fig. 2). The stronger the mutant allele, the more compact the spike. As discussed by Nanape *et al.* (2024), extremely compact spikes are undesirable in practical cultivars. In contrast, CL barley cultivars have slightly reduced spikes with no significant disadvantages because the introduced *cly1.b* or *cly1.c* mutant alleles have a mild effect. This is consistent with predictions based on their interactions with miR172 (Nanape *et al.* 2023) and may reflect synonymous substitutions. This is in marked contrast to other induced barley *HvAP2* mutants, which harbor distinct point mutations at the miR172 target site. Although all these mutants exhibit cleistogamy, their spikes are much more compact than those of *cly1.b* or *cly1.c* (Houston *et al.* 2013). Therefore, a direct strategy for developing CL wheat with non-compact spikes is to combine *AP2* homoeologous mutant alleles carrying the same point mutations found in the *cly1.b* or *cly1.c* alleles of barley. These point mutations can be introduced using genome-editing techniques designed for single-base replacements. This precise base substitution has been successfully applied in rice to edit a miR172 target site in the *OsCly1* gene (Nishizawa-Yokoi *et al.* 2015, Ohtsuki *et al.* 2021). Another interesting approach is to modify the expression levels of *AP2* homoeologs independently of the miRNA pathway. Altering the promoter or gene regulatory elements of mutant *AP2* alleles could increase or decrease their expression. This would enable proper control of lodicule suppression and spike compactness. Furthermore, this approach is not limited to mutant alleles with a point mutation in the miR172 target site. According to the dosage mechanism, the wild-type *AP2* allele would function if its expression level could be increased. Overall, these approaches present intriguing challenges for improving cleistogamy traits in wheat breeding and further elucidating the dosage mechanisms of wheat *AP2* homoeologs.

## Author Contribution Statement

KK designed the study. KK, MN and ABN were responsible for generating plant populations, genotyping and qPCR assays. ABN and HO conducted the field survey, performed the microscopy and analyzed the data. KK wrote the manuscript, which was approved by all authors.

## Supplementary Material

Supplemental Figures

Supplemental Tables

## Figures and Tables

**Fig. 1.**
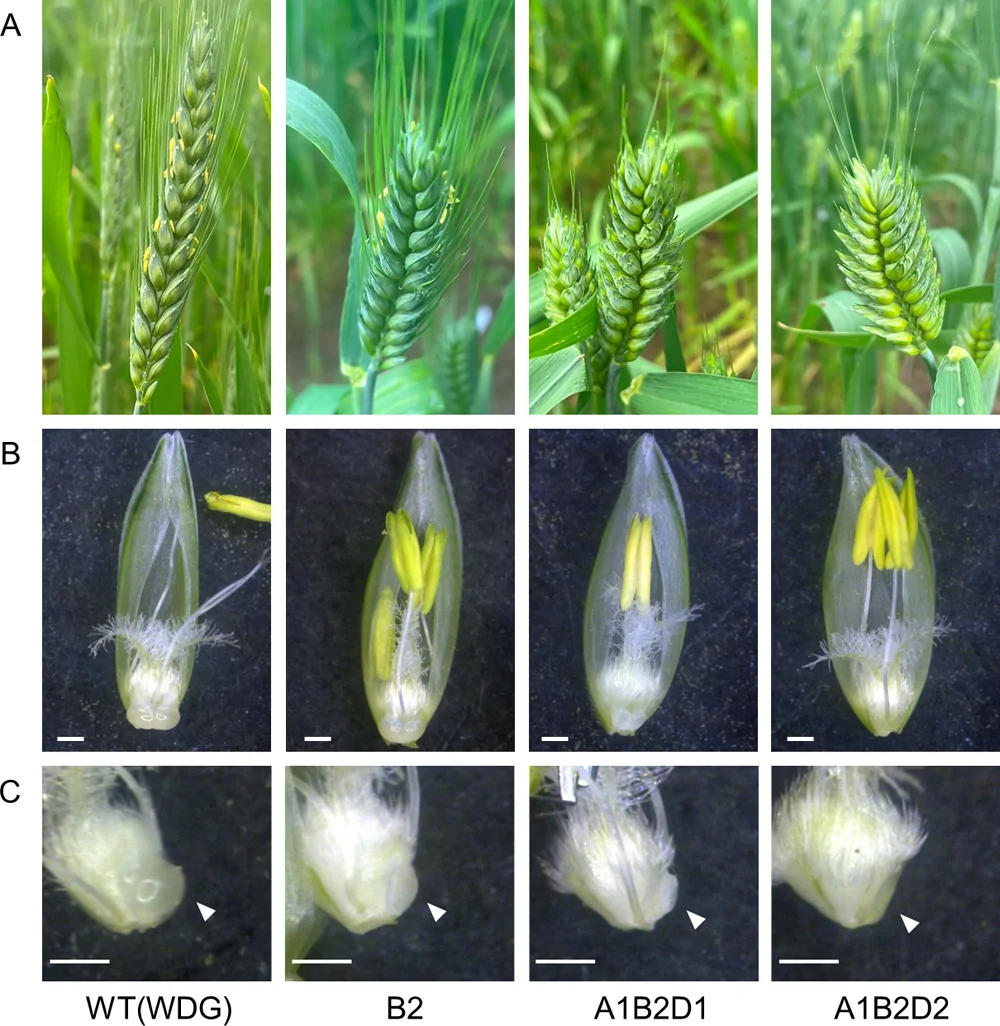
Spikes (A), florets (B) and lodicules (C) at anthesis. The following four genotypes are shown: cv. ‘Wedgetail’(WT(WDG)), the single mutant *AP2-B2* (B2), and the two triple mutants (A1B2D1 and A1B2D2). The lemma has been removed in (B). Side views of the enlarged lodicules (indicated by arrowheads) are shown in (C), with the lemmas and paleas removed. Bar: 1 mm.

**Fig. 2.**
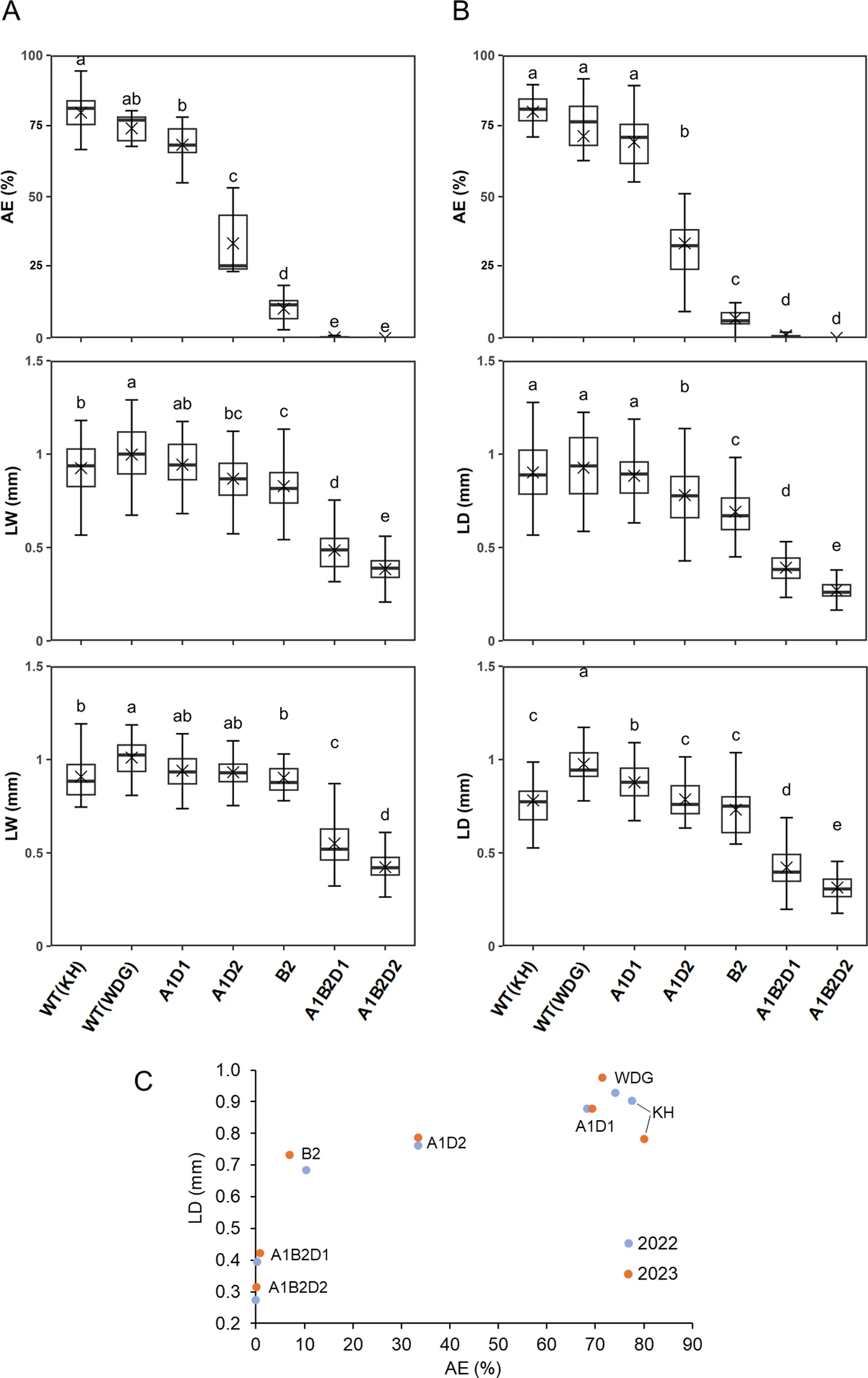
Comparison of anther extrusion (AE) rate and lodicule size (lodicule width (LW) and lodicule depth (LD)) among the seven genotypes shown in Table 1. (A) The 2022 data, including the two triple mutants in the F_2_ generation. (B) The 2023 data, including the two triple mutants in the F_3_ generation. (C) Scattergram of AE and LD for the mean values in 2022 and 2023. In box plots A and B, thick horizontal lines indicate median (50% interquartile range), crosses indicate mean, and whiskers indicate maximum and minimum values excluding outliers. According to the Tukey–Kramer HSD test, values with the same letter do not differ significantly (*P* > 0.05). Sample sizes (*n*, number of lodicules) are shown in Supplemental Table 2.

**Fig. 3.**
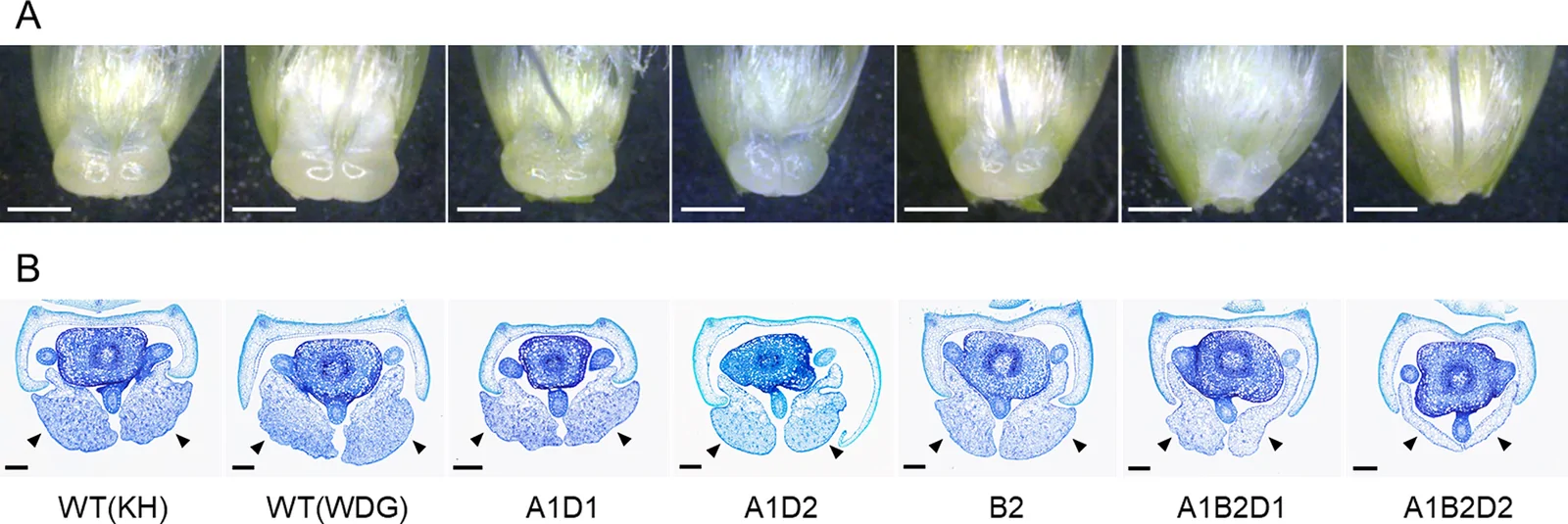
Lodicules at anthesis. (A) Enlarged micrographs of a pair of lodicules in a floret. (B) Transverse sections of a floret. Arrowheads indicate the lodicules. The lemma has been removed in (A) and (B). Seven genotypes are shown for comparison; see Table 1 for their abbreviations. Bar: 1 mm (A); 0.2 mm (B).

**Fig. 4.**
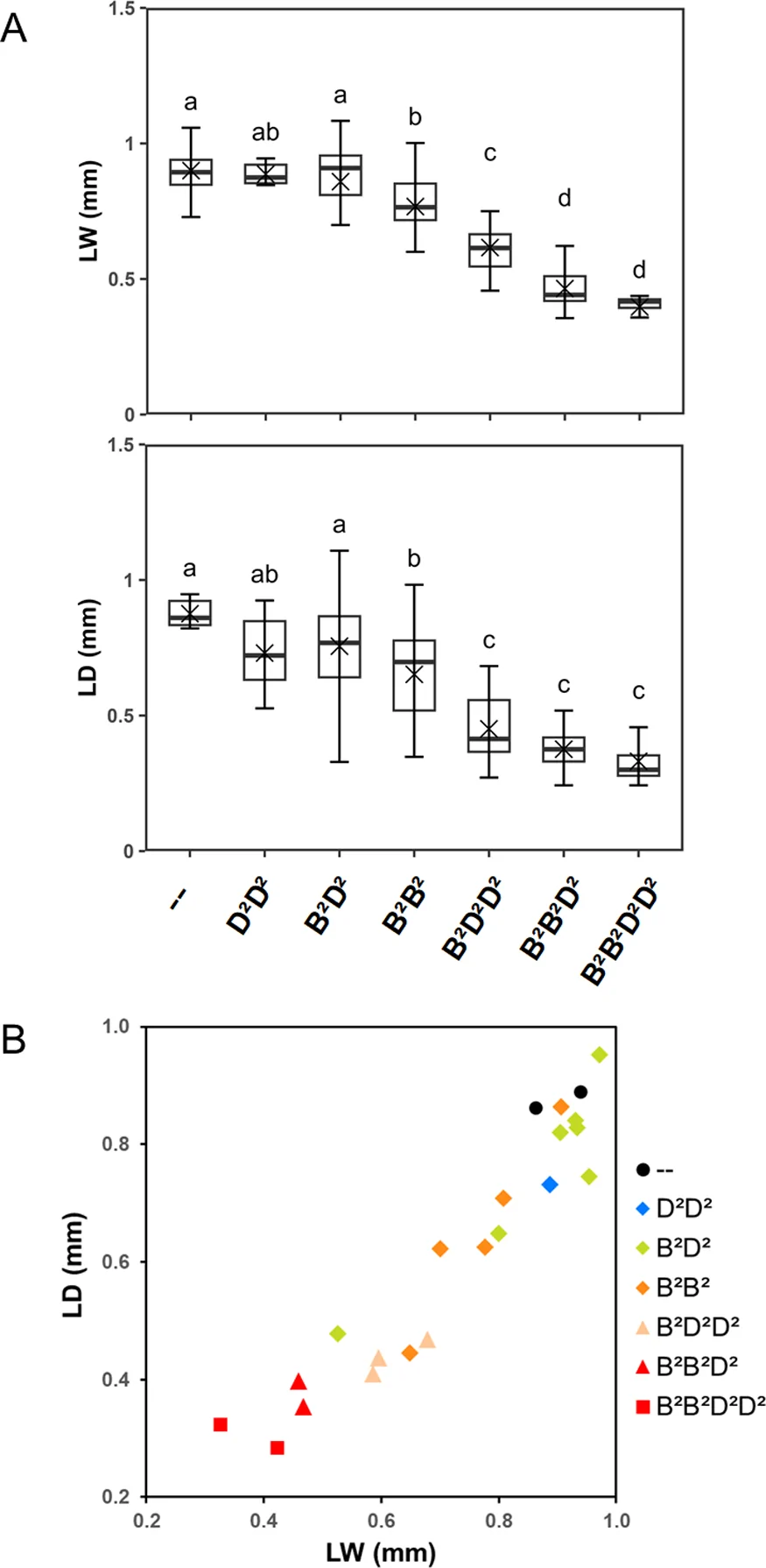
Comparison of lodicule size among F_2_ (B2 × D2) segregating genotypes. (A) Lodicule width (LW) and lodicule depth (LD). (B) Scattergram of LW and LD. Each genotype is abbreviated to include only the mutant alleles (*B^2^* and *D^2^*) that it carries, and the wild-type genotype (*BBDD*) is indicated using -- (see Table 2). In the box plots, thick horizontal lines indicate median (50% interquartile range), crosses indicate mean, and whiskers indicate maximum and minimum values excluding outliers. According to the Tukey–Kramer HSD test, values with the same letter do not differ significantly (*P* > 0.05). Sample sizes (*n*, number of lodicules) are shown in Supplemental Table 2. The scattergram plots the mean values of each F_2_ plant.

**Fig. 5.**
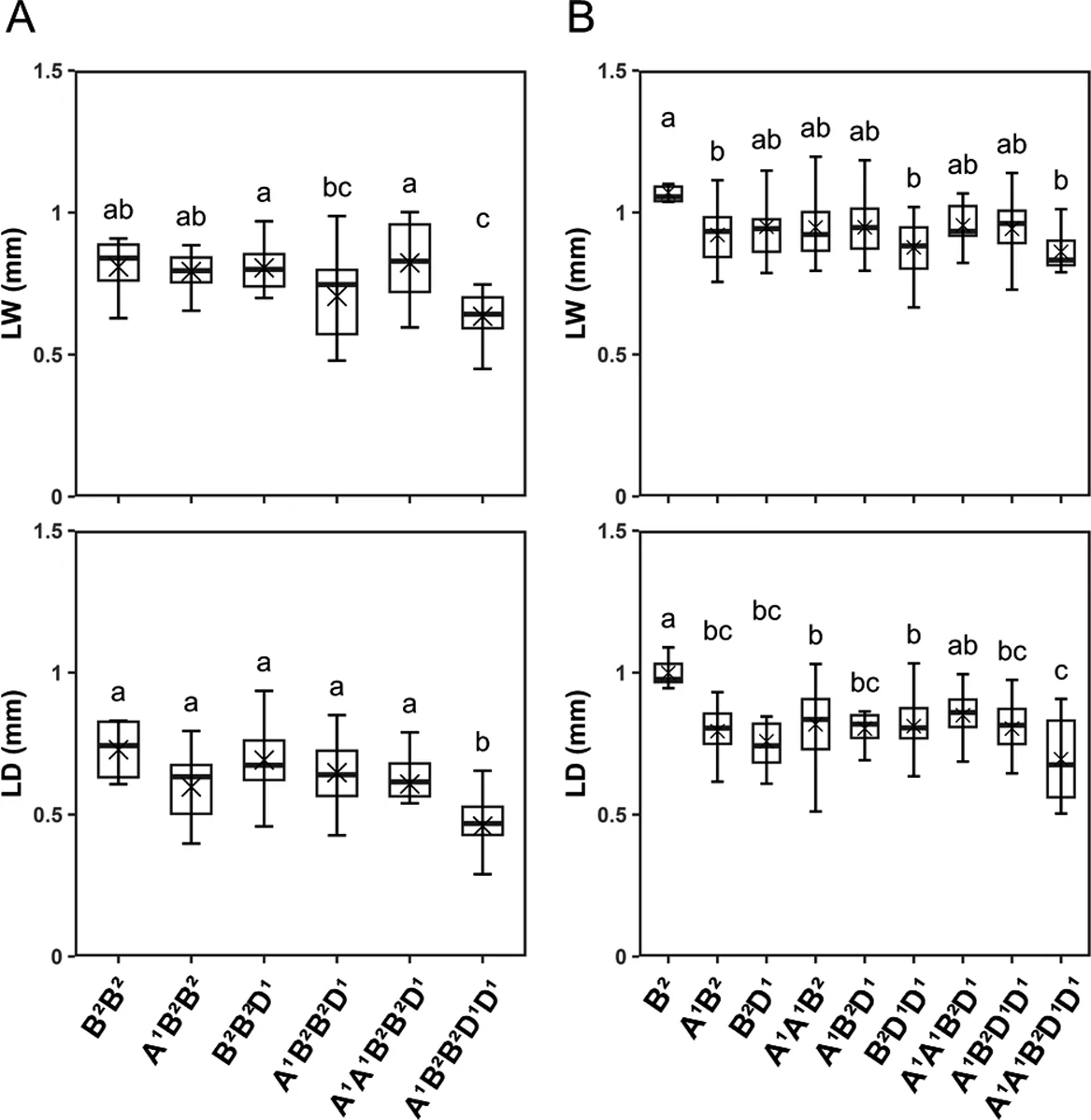
Comparison of lodicule size among F_2_ (A1D1 × B2) segregating genotypes. (A) Six genotypes that carry two mutant *B^2^* alleles. (B) Nine genotypes that carry one mutant *B^2^* allele. Lodicule width (LW, left) and depth (LD, right) are shown. Each genotype is abbreviated to include only the mutant alleles (*B^2^*, *A^1^* and *D^1^*) that it carries (see Table 2). In the box plots, thick horizontal lines indicate median (50% interquartile range), crosses indicate mean, and whiskers indicate maximum and minimum values excluding outliers. According to the Tukey–Kramer HSD test, values with the same letter do not differ significantly (*P* > 0.05). Sample sizes (*n*, number of lodicules) are shown in Supplemental Table 2.

**Fig. 6.**
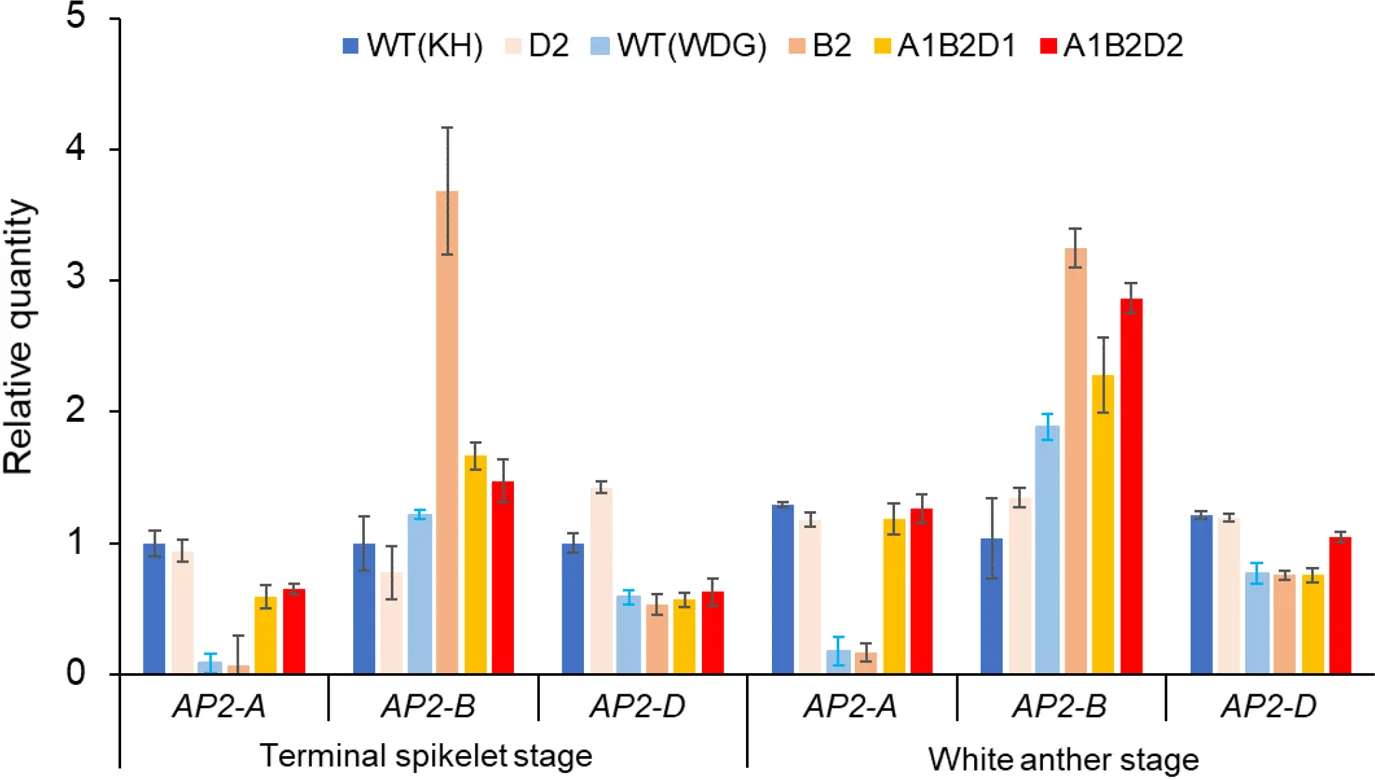
Transcript abundance of three *AP2* homoeologs in immature spikes at the terminal spikelet and white anther stages. qPCR was performed for each *AP2* homoeolog in six genotypes: two wild-type cultivars (WT(KH) and WT(WDG)), two single mutants (D2 and B2) and two triple mutants (A1B2D1 and A1B2D2). Transcript abundances were normalized to the levels of *actin* and WT(KH) at the terminal spikelet stage. Each genotype was assayed as two biological replicates.

**Table 1.** A list of seven genotypes, including two triple mutants and their parental genotypes, along with their abbreviations and flowering types

Geneotype	Abbreviation	*AP2* genotype in short allele symbols	Flowering type*^a^*
cv. ‘Kitahonami’	WT(KH)	*AA BB DD*	CH
cv. ‘Wedgetail’	WT(WDG)	*AA BB DD*	CH
Double mutant *AP2-A1/AP2-D1*	A1D1	*A^1^A^1^ BB D^1^D^1^*	CH
Double mutant *AP2-A1/AP2-D2*	A1D2	*A^1^A^1^ BB D^2^D^2^*	CH
Single mutant *AP2-B2*	B2	*AA B^2^B^2^ DD*	CH
Triple mutant *AP2-A1/AP2-B2/AP2-D1*	A1B2D1	*A^1^A^1^ B^2^B^2^ D^1^D^1^*	CL
Triple mutant *AP2-A1/AP2-B2/AP2-D2*	A1B2D2	*A^1^A^1^ B^2^B^2^ D^2^D^2^*	CL

*^a^* CH: normal chasmogamous; CL: cleistogamous.

**Table 2.** Segregating genotypes and their flowering types in two F_2_ populations

Line	Genotype	Abbreviation	*n*	Flowering type^*a*^
F_2_ (B2 × D2)	*BB DD*	—	5	CH
	*BB DD^2^*	*D^2^*	7	CH
	*BB^2^ DD*	*B^2^*	9	CH
	*BB D^2^D^2^*	*D^2^D^2^*	4	CH
	*BB^2^ DD^2^*	*B^2^D^2^*	11	CH
	*B^2^B^2^ DD*	*B^2^B^2^*	8	CH
	*BB^2^ D^2^D^2^*	*B^2^D^2^D^2^*	9	CL
	*B^2^B^2^ DD^2^*	*B^2^B^2^D^2^*	6	CL
	*B^2^B^2^ D^2^D^2^*	*B^2^B^2^D^2^D^2^*	3	CL
F_2_ (A1D1 × B2)	*AA BB DD*	---	2	CH
	*AA^1^ BB DD*	*A^1^*	0	n/a
	*AA BB DD^1^*	*D^1^*	2	CH
	*AA BB^2^ DD*	*B^2^*	5	CH
	*A^1^A^1^ BB DD*	*A^1^A^1^*	2	CH
	*AA^1^ BB DD^1^*	*A^1^D^1^*	6	CH
	*AA BB D^1^D^1^*	*D^1^D^1^*	1	CH
	*AA^1^ BB^2^ DD*	*A^1^B^2^*	8	CH
	*AA BB^2^ DD^1^*	*B^2^D^1^*	3	CH
	*AA B^2^B^2^ DD*	*B^2^B^2^*	2	CH
	*A^1^A^1^ BB DD^1^*	*A^1^A^1^D^1^*	2	CH
	*AA^1^ BB D^1^D^1^*	*A^1^D^1^D^1^*	5	CH
	*A^1^A^1^ BB^2^ DD*	*A^1^A^1^B^2^*	7	CH
	*AA^1^ BB^2^ DD^1^*	*A^1^B^2^D^1^*	5	CH
	*AA BB^2^ D^1^D^1^*	*B^2^D^1^D^1^*	4	CH
	*AA^1^ B^2^B^2^ DD*	*A^1^B^2^B^2^*	4	(CH)
	*AA B^2^B^2^ DD^1^*	*B^2^B^2^D^1^*	1	CH
	*A^1^A^1^ BB D^1^D^1^*	*A^1^A^1^D^1^D^1^*	3	CH
	*A^1^A^1^ BB^2^ DD^1^*	*A^1^A^1^B^2^D^1^*	3	CH
	*AA^1^ BB^2^ D^1^D^1^*	*A^1^B^2^D^1^D^1^*	8	CH
	*A^1^A^1^ B^2^B^2^ DD*	*A^1^A^1^B^2^B^2^*	0	n/a
	*AA^1^ B^2^B^2^ DD^1^*	*A^1^B^2^B^2^D^1^*	8	(CH)
	*AA B^2^B^2^ D^1^D^1^*	*B^2^B^2^D^1^D^1^*	2	(CH)
	*A^1^A^1^ BB^2^ D^1^D^1^*	*A^1^A^1^B^2^D^1^D^1^*	2	CH
	*A^1^A^1^ B^2^B^2^ DD^1^*	*A^1^A^1^B^2^B^2^D^1^*	2	CL
	*AA^1^ B^2^B^2^ D^1^D^1^*	*A^1^B^2^B^2^D^1^D^1^*	5	CL
	*A^1^A^1^ B^2^B^2^ D^1^D^1^*	*A^1^A^1^B^2^B^2^D^1^D^1^*	0	n/a

*n*: number of plants observed for flowering type in the field. *^a^* CH: normal chasmogamous; (CH): chasmogamous, but with highly suppressed anther extrusion; CL: cleistogamous; n/a: not available.
