# Supplementary material for: Wheat cleistogamy is expressed by the dosage effect of homoeologous AP2 mutant alleles carrying a point mutation in the miR172 target site
Source: Breed Sci. 2026 Jun 23;76(3):299–308. doi: 10.1270/jsbbs.25078 (PMC13709628; doi:10.1270/jsbbs.25078)
Supplement: Supplementary file 1 [file 76_299_s1.pdf]

doi: 10.1270/jsbbs.25078

| | | 5' | miR172 target site | | | | | | | 3' |
|---|---|---|---|---|---|---|---|---|---|---|
| Wild type | *AP2-A* | ... | CT | GCA | GCA | TCA | TCA | CGA | TTC | C ... |
| | *AP2-B* | ... | CC | GCA | GCA | TCA | TCA | CGA | TTC | C ... |
| | *AP2-D* | ... | CT | GCA | GCA | TCA | TCA | CGA | TTC | C ... |
| Mutant | *AP2-A1* | ... | CT | GCA | G**T**A | TCA | TCA | CGA | TTC | C ... |
| | *AP2-D1* | ... | CT | GCA | G**T**A | TCA | TCA | CGA | TTC | C ... |
| | *AP2-D2* | ... | CT | GCA | **A**CA | TCA | TCA | CGA | TTC | C ... |
| | *AP2-B2* | ... | CC | GCA | GCA | TCA | TCA | C**A**A | TTC | C ... |

Supplemental Fig. 1. Sequence variation in the miR172 target sites of the three *AP2* homoeologs in hexaploid wheat. Point mutations in the mutant alleles are indicated in red. The *AP2-B2* sequence was obtained from Debernardi *et al.* (2020), and the other sequences were obtained from Nanape *et al.* (2023).

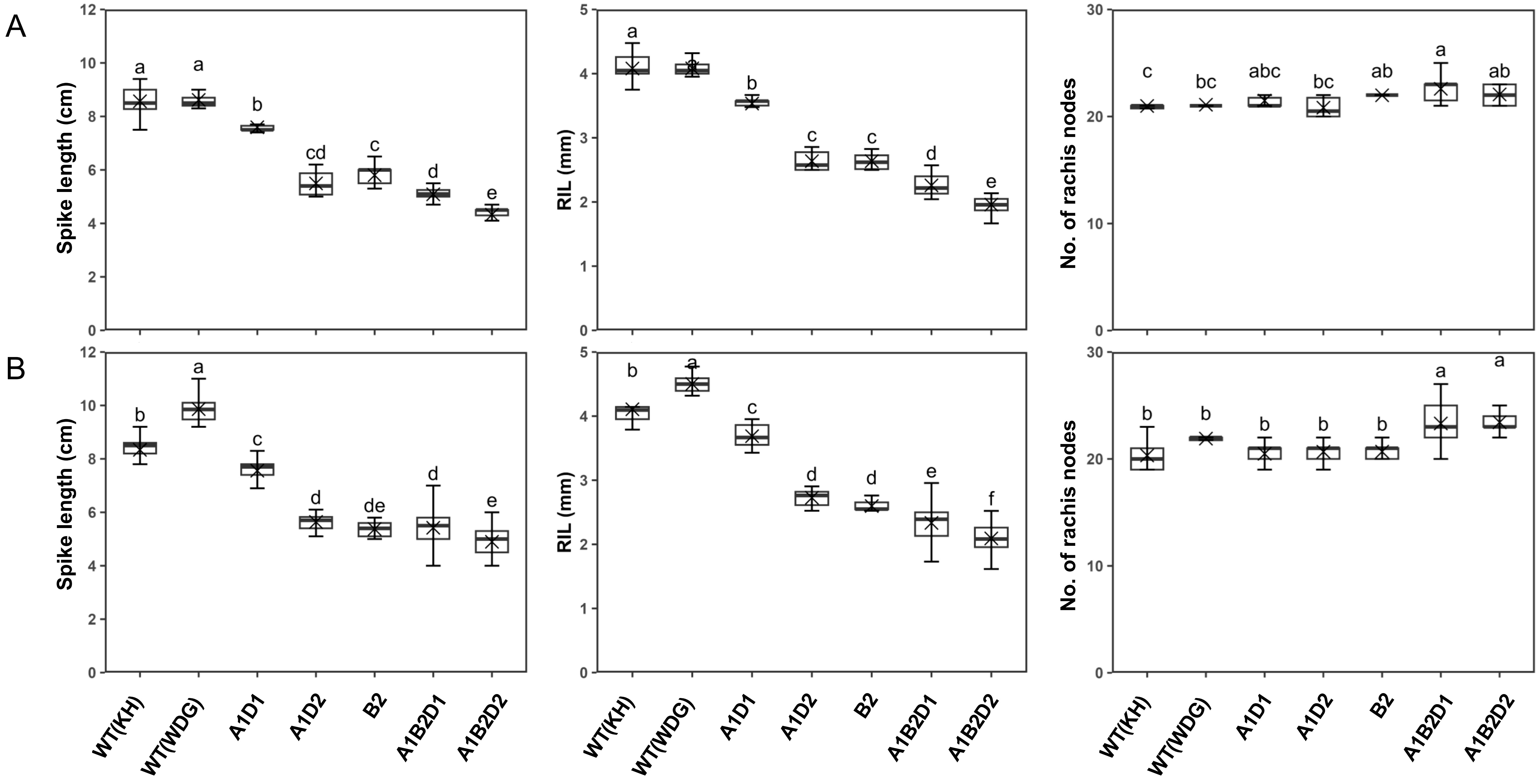


Supplemental Fig. 2. Comparison of spike (rachis) length (left), rachis internode length (RIL, center), and number of rachis nodes (right) among the seven genotypes. (A) 2022 data points and (B) 2023 data points. Refer to the legends of Fig. 2 for explanations of the box plots.

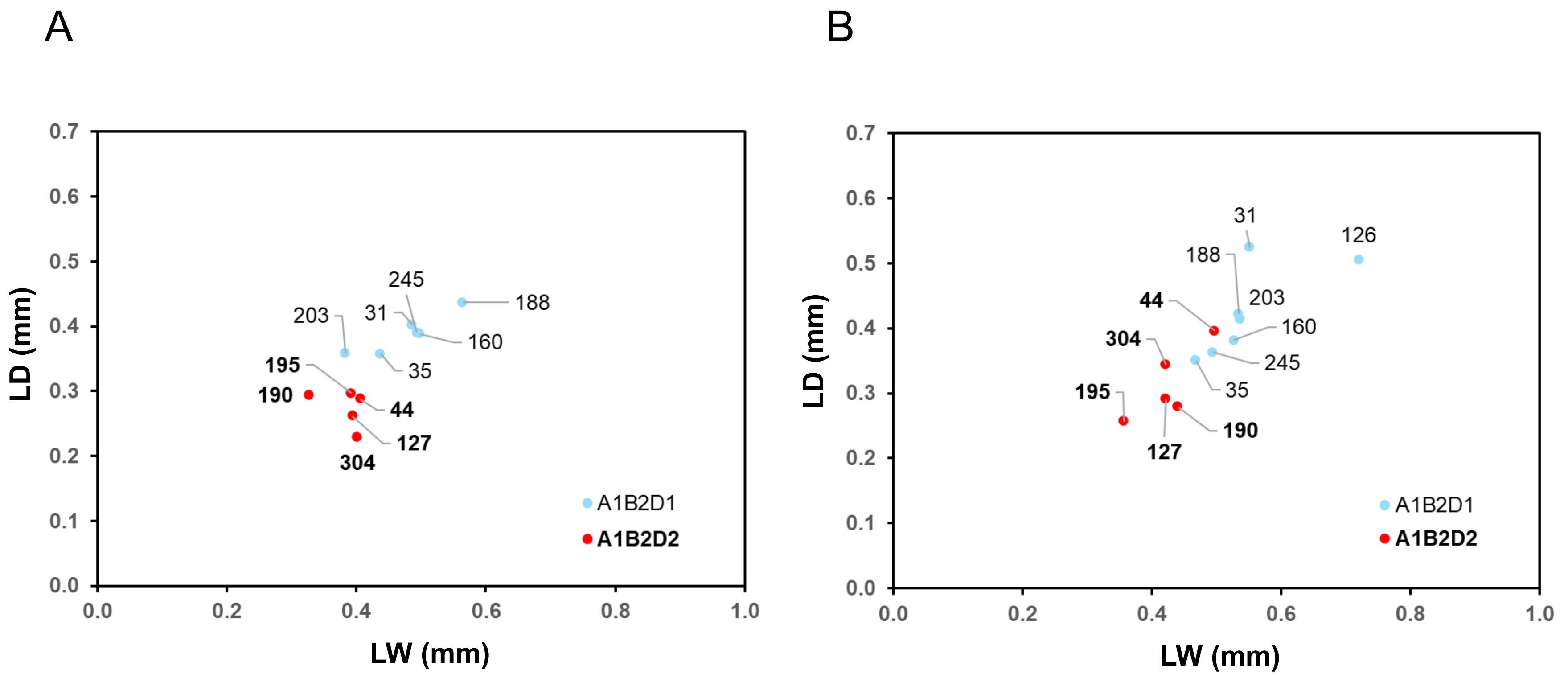


Supplemental Fig. 3. Scattergrams of LW and LD for the two triple mutants. (A) 2022 data points and (B) 2023 data points. The mean values for each $F_2$ plant (A) and each $F_3$ progeny derived from an $F_2$ plant (B) are plotted along with the corresponding $F_2$ plant number.

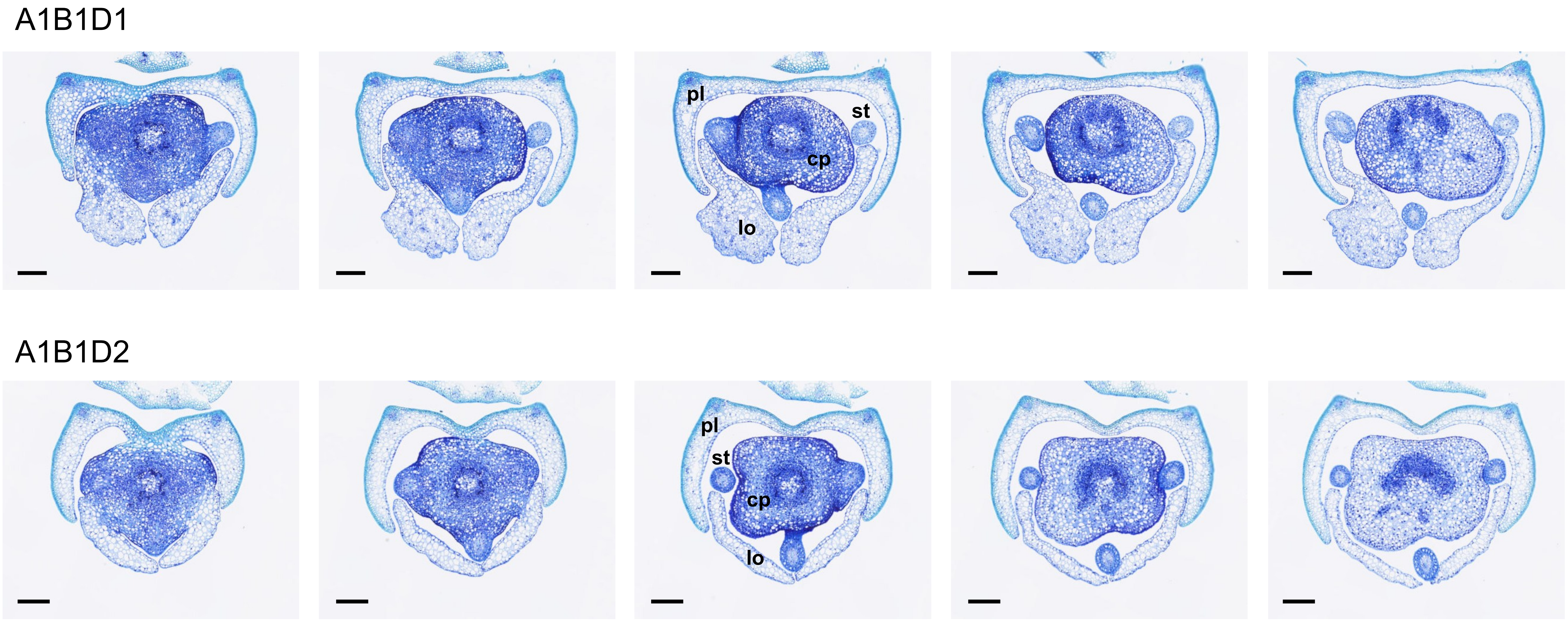


Supplemental Fig. 4. Transverse sections of a floret at anthesis in two triple mutants: A1B2D1 (top panel) and A1B2D2 (bottom panel). Serial sections of the lodicules from the lower to the upper parts are shown from left to right. The lemma has been removed. pl: palea, st: stamen, lo: lodicule, cp: carpel. Bar: 0.2 mm.

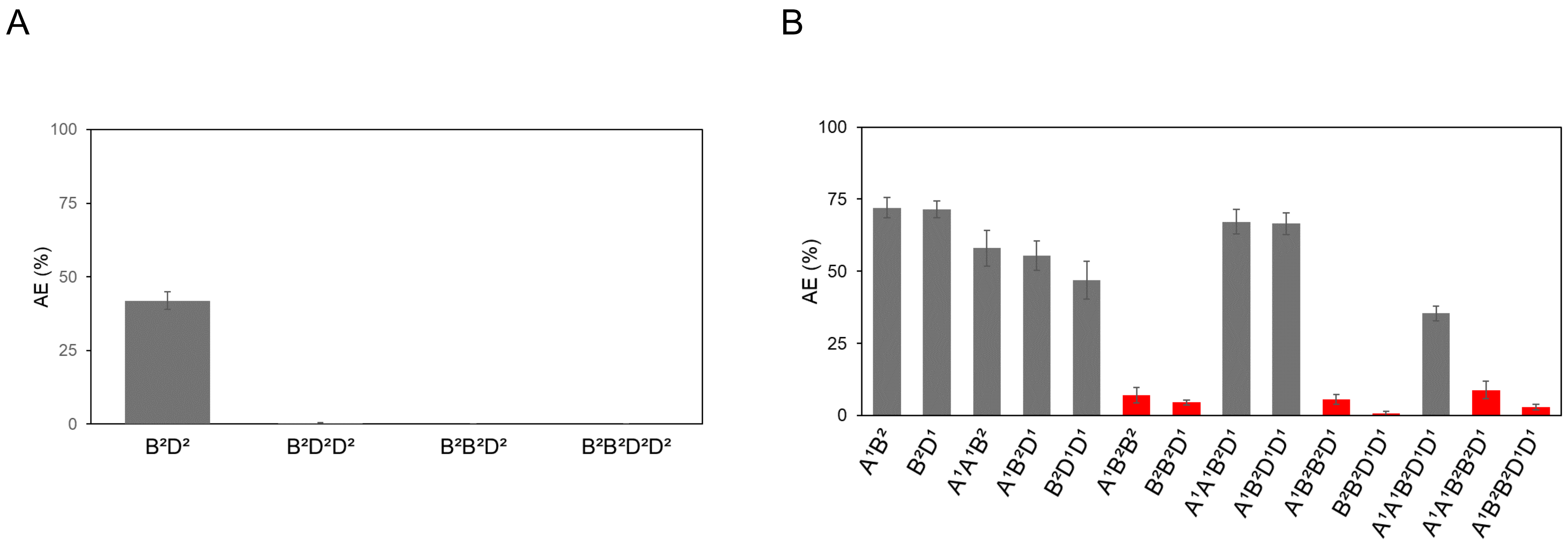


Supplemental Fig. 5. Comparison of the anther extrusion (AE) rates. (A) Four genotypes were examined in the $F_2$ (B2 × D2) population. (B) Fourteen genotypes from the $F_2$ (A1D1 × B2) population were examined. Genotypes carrying two $B^2$ alleles are indicated in red. See Tables 1 and 2 for details on the genotype designation. Means and standard error (SE) bars are shown.

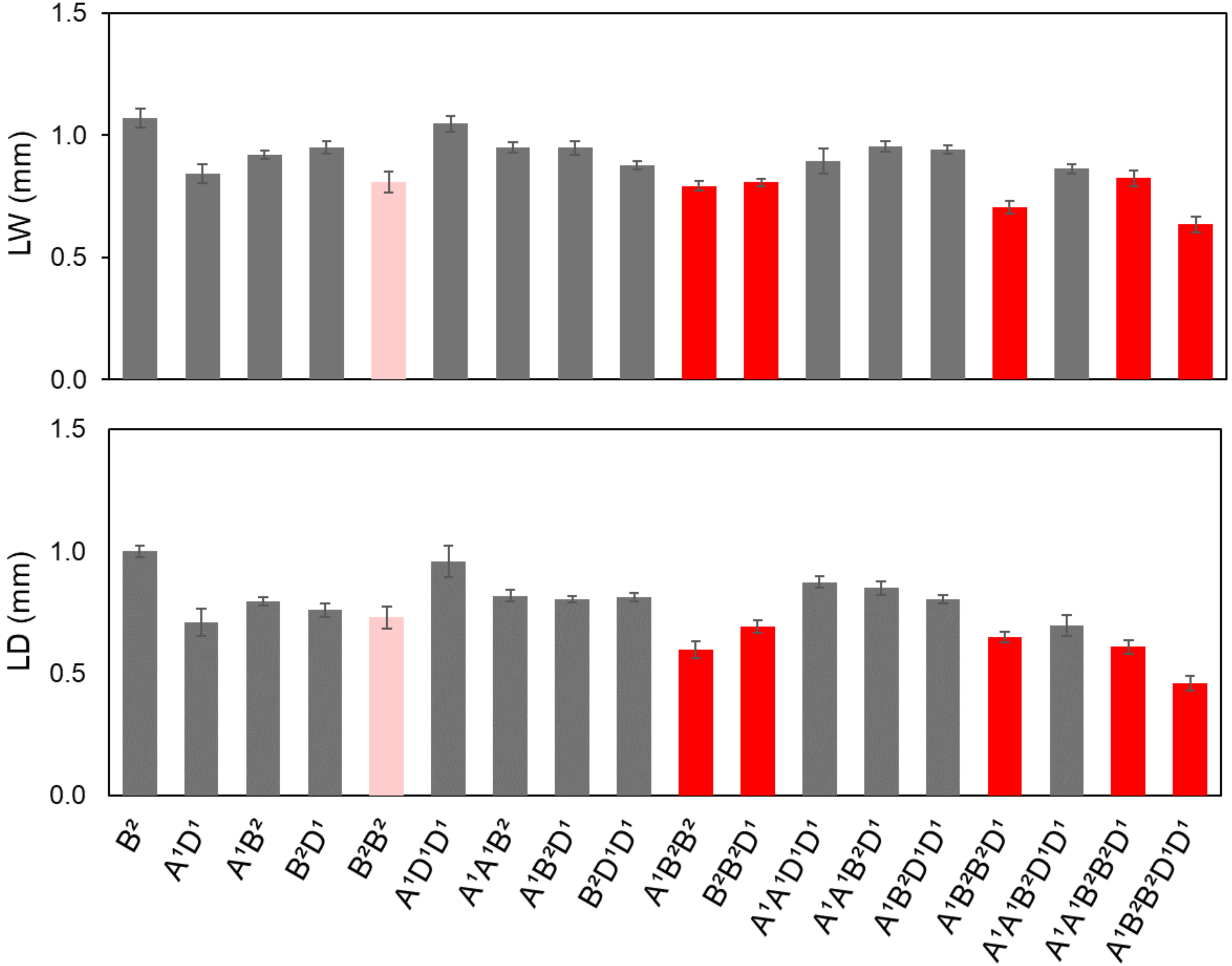


Supplemental Fig. 6. Comparison of lodicule sizes among the 18 genotypes examined in the $F_2$ (A1D1 × B2) population. The LW (top) and LD (bottom) are shown as means with SE bars. Genotypes carrying the two $B^2$ alleles are highlighted. See Table 2 for genotype abbreviations.
