# Supplementary material for: Wheat cleistogamy is expressed by the dosage effect of homoeologous AP2 mutant alleles carrying a point mutation in the miR172 target site
Source: Breed Sci. 2026 Jun 23;76(3):299–308. doi: 10.1270/jsbbs.25078 (PMC13709628; doi:10.1270/jsbbs.25078)
Supplement: Supplementary file 2 [file 76_299_s2.pdf]

doi: 10.1270/jsbbs.25078

Supplemental Table 1. Primers and PCR conditions used to distinguish between the wild-type and mutant *AP2-B2* alleles

**Target**	**Primer**	**Sequence (5' to 3`)**	**PCR conditions**
AP2-B	TaAP2B-KASP_Wt2	AGCCGCAGCATCATCACG	95° C/2 min, (95° C/30 s, 64° C/30 s, 72° C/30 s) × 30 cycles
Wild type	Rv-TaAP2B1	GACGGATGGTCTCCGGGTAC	
AP2-B2	TaAP2B-KASP_Mut2	AGCCGCAGCATCATCACA	95° C/2 min, (95° C/30 s, 64° C/30 s, 72° C/30 s) × 30 cycles
Mutant	Rv-TaAP2B1	GACGGATGGTCTCCGGGTAC	

* Each 10 µL PCR mixture contains 0.25 U ExTaq DNA polymerase (Takara Bio Inc., Ōtsu, Japan), 1 × Ex Taq polymerase buffer, 8% DMSO, 0.3µM of each primer, and template DNA.

Supplemental Table 2. Number of lodicules or spikes (*n*) used to measure lodicule size (LW and LD) or AE rate

A. Fig. 2A

Genotype	*n*	
	AE rate	LW, LD
WT(KH)	20	68
WT(WDG)	9	48
A1D1	6	48
A1D2	6	48
B2	15	56
A1B2D1	15	72
A1B2D2	13	52

B. Fig. 2B

Genotype	*n*	
	AE rate	LW, LD
WT(KH)	9	34
WT(WDG)	12	18
A1D1	18	34
A1D2	16	36
B2	10	18
A1B2D1	39	148
A1B2D2	41	108

C. Fig. 4A

Genotype	*n*	
	LW	LD
--	12	12
D^2D^2	6	6
B^2D^2	42	42
B^2B^2	30	30
$B^2D^2D^2$	18	18
$B^2B^2D^2$	30	30
$B^2B^2D^2D^2$	8	12

D. Fig. 5A

Genotype	*n*
	LW, LD
B^2B^2	6
$A^1B^2B^2$	12
$B^2B^2D^1$	22
$A^1B^2B^2D^1$	30
$A^1A^1B^2B^2D^1$	18
$A^1B^2B^2D^1D^1$	14

E. Fig. 5B

Genotype	*n*
	LW, LD
B^2	6
A^1B^2	30
B^2D^1	18
$A^1A^1B^2$	22
$A^1B^2D^1$	16
$B^2D^1D^1$	30
$A^1A^1B^2D^1$	12
$A^1B^2D^1D^1$	30
$A^1A^1B^2D^1D^1$	12
